# Haplogroup T Is an Obesity Risk Factor: Mitochondrial DNA Haplotyping in a Morbid Obese Population from Southern Italy

**DOI:** 10.1155/2013/631082

**Published:** 2013-07-02

**Authors:** Carmela Nardelli, Giuseppe Labruna, Rosario Liguori, Cristina Mazzaccara, Maddalena Ferrigno, Valentina Capobianco, Massimo Pezzuti, Giuseppe Castaldo, Eduardo Farinaro, Franco Contaldo, Pasqualina Buono, Lucia Sacchetti, Fabrizio Pasanisi

**Affiliations:** ^1^CEINGE Biotecnologie Avanzate S.C. a R.L., Via G. Salvatore 486, 80145 Naples, Italy; ^2^Dipartimento di Medicina Molecolare e Biotecnologie Mediche, Università degli Studi di Napoli Federico II, Via S. Pansini 5, 80131 Naples, Italy; ^3^IRCCS Fondazione SDN, Istituto di Ricerca Diagnostica e Nucleare, Via E. Gianturco 113, 80143 Naples, Italy; ^4^Dipartimento di Sanità Pubblica, Università degli Studi di Napoli Federico II, Via S. Pansini 5, 80131 Naples, Italy; ^5^CISRO, Centro Interuniversitario di Studi e Ricerche sull'Obesità e Dipartimento di Medicina Clinica e Chirurgia, Università degli Studi di Napoli Federico II, Via S. Pansini 5, 80131 Naples, Italy; ^6^Dipartimento di Studi delle Istituzioni e dei Sistemi Territoriali, Università degli Studi “Parthenope”, Via F. Acton 38, 80133 Naples, Italy; ^7^Laboratori Misti, IRCCS Fondazione SDN-CEINGE, Via E. Gianturco 113, 80143 Naples, Italy

## Abstract

Mitochondrial DNA (mtDNA) haplogroups have been associated with the expression of mitochondrial-related diseases and with metabolic alterations, but their role has not yet been investigated in morbid obese Caucasian subjects. Therefore, we investigated the association between mitochondrial haplogroups and morbid obesity in patients from southern Italy. The mtDNA D-loop of morbid obese patients (*n* = 500; BMI > 40 kg/m^2^) and controls (*n* = 216; BMI < 25 kg/m^2^) was sequenced to determine the mtDNA haplogroups. The T and J haplogroup frequencies were higher and lower, respectively, in obese subjects than in controls. Women bearing haplogroup T or J had twice or half the risk of obesity. Binomial logistic regression analysis showed that haplogroup T and systolic blood pressure are risk factors for a high degree of morbid obesity, namely, BMI > 45 kg/m^2^ and in fact together account for 8% of the BMI. In conclusion, our finding that haplogroup T increases the risk of obesity by about two-fold, suggests that, besides nuclear genome variations and environmental factors, the T haplogroup plays a role in morbid obesity in our study population from southern Italy.

## 1. Introduction

Obesity is a multifactorial disorder caused by a combination of environmental, behavioural, and genetic factors; however, the molecular mechanisms by which these factors provoke fat mass accumulation and maintenance are not yet completely elucidated [1]. Moreover, mitochondrial dysfunctions that result in lipid accumulation and insulin resistance have been implicated in the pathogenesis of obesity [2]. Several mitochondrial DNA (mtDNA) variants have been investigated in diverse populations with obesity-related and lipid metabolism alterations [3, 4], and it was suggested that particular mtDNA haplogroups could be associated with inefficient energy expenditure [5]. A mitochondrial haplogroup (mt-haplogroup) is a collection of single nucleotide polymorphisms [6] accumulated throughout human history in specific populations that could be attributed to genetic drift and/or climate selection [7–9]. Mitochondrial haplogroups have been associated with the expression of mitochondrial-related diseases (metabolic syndrome, type 2 diabetes, neurological disorders, infertility, and Parkinson's disease) and with various individual characteristics (aging and endurance training capacity) [3, 10–14], but, to our knowledge, their role has not yet been investigated in morbid obese Caucasian subjects. We previously reported that several DNA variants and epigenetic alterations are associated with the obese phenotype and/or with obese-related diseases [15–19]. Here, we have characterized mt-haplogroups in a large population of morbid obese adults and nonobese individuals from southern Italy to look for associations among specific mt-haplogroups and the obese phenotype.

## 2. Methods

### 2.1. Study Population

Five hundred unrelated morbid obese patients (64% females, age range 17–70 years, median; 2.5th–97.5th percentiles BMI = 45.1; 38.2–65.4 kg/m^2^), and 216 non obese controls (66% females, age range 26–76 years, BMI = 22.9; 18.2–25.6 kg/m^2^) were recruited from the Obesity Outpatient Clinic of the Dipartimento di Medicina Clinica e Chirurgia and from the Dipartimento di Medicina Molecolare e Biotecnologie Mediche, Federico II University Hospital, Naples (Italy), respectively. All participants were Caucasians and had lived in southern Italy for at least 3 generations. Written informed consent for participation in the study was obtained from all subjects. The research was approved by the Ethics Committee of the School of Medicine, University of Naples Federico II, and was in accordance with the principles of the Helsinki II Declaration.

### 2.2. Laboratory Investigations

Two blood samples (one for biochemical analysis and one for DNA extraction) were obtained after an overnight fast from each enrolled subject. Biochemical parameters were measured enzymatically with routine methods on an automated analyzer (Hitachi 747; Boehringer Mannheim, Germany). Insulin resistance was estimated in obese subjects with the homeostasis model assessment (HOMA) and the formula: fasting insulin (mU/L) X fasting glucose (mmol/L)/22.5. 

The clinical and anamnestic data of each obese subject were collected, and the main metabolic parameters were evaluated. Fat mass (FM) and fat-free mass (FFM) were measured by bioimpedentiometry (Sta/BIA Akern, Florence, Italy) and respiratory quotient (RQ) and basal metabolic rate (BMR) by indirect calorimetry (Sensor Medics Vmax29, Anaheim, CA, USA). The BMI was calculated as weight (kg)/square height (m^2^). Systolic and diastolic blood pressure, and cardiac frequency (beats/min) were collected by standard procedures. Metabolic syndrome (MS), which is a cluster of metabolic risk factors, namely, abdominal obesity, dyslipidemia (hypertriglyceridemia or low HDL-cholesterol concentrations), elevated blood pressure, and hyperglycemia, as defined by the American Heart Association criteria, was diagnosed if 3 out of the 5 risk factors were present [20].

### 2.3. Mitochondrial DNA Amplification and Sequencing

Genomic DNA was extracted from whole blood (Illustra BACC-2; GE Healthcare, UK). The D-loop region (about 1100 bp) was amplified with primers (HVI-forward: GTAAACCGGAGATGAAAACCT; HVII-reverse ACTGCATACCGCCAAAAGATA) chosen by the PRIMER 3 program (http://frodo.wi.mit.edu/) in a final volume of 25 *μ*L containing a PCR mixture (10 *μ*M each primer, 10x PCR buffer, 200 *μ*M each deoxynucleotide triphosphate, and 0.5 U of Taq DNA polymerase) and 50 ng of genomic DNA. PCR consisted of an initial denaturation at 95°C for 5 min, followed by 35 cycles of 95°C for 30 s, 60°C for 1 min, and 72°C for 1 min, followed by a final extension at 72°C for 5 min. The PCR fragment was run on a 1.5% agarose gel, purified, and sequenced in both directions (BigDye Terminator v3.1 cycle sequencing method on an ABI-Prism 3730 DNA Analyzer; Applied Biosystems). Sequences were assembled with the SeqScape program v.2.5. The mtDNA nucleotide sequences were numbered according to the rCRS reference sequence (NC_012920). Haplogroups were defined by nucleotides at specific known polymorphic sites in the mtDNA [6].

### 2.4. Statistical Analysis

Haplogroup frequencies in patients and control subjects were compared using the *χ* ^2^ test. A *P* < 0.05 was considered the level of statistical significance. The odds ratio (OR) and 95% confidence intervals (CI) values for each haplogroup were calculated for the statistically significant haplogroup. ANOVA was performed to compare metabolic parameters in the different haplogroups. Multiple comparisons were corrected with the Bonferroni test. The Student's *t*-test, followed by a binomial logistic regression analysis, was used to investigate the association between the biochemical and clinical characteristics and the presence/absence of a specific haplogroup. Sample estimates were verified by bootstrapping. Statistical analyses were carried out with the PASW package for Windows (Ver.18; SPSS Inc. Headquarters, Chicago, Il, USA).

## 3. Results

The clinical and biochemical characteristics of the obese subjects are reported in Table 1. Metabolic syndrome was present in 41.3% of our obese subjects and was significantly more frequent in men than in women (47.9% versus 37.5%; OR/95% CI = 1.17/1.01–1.35, *P* = 0.031). Concurrent MS factors in our obese patients were hypertriglyceridemia (OR/95% CI: 16.7/10.8–26.0), hyperglycemia (OR/95% CI: 8.5/5.6–12.8), hypertension (OR/95% CI: 7.1/4.7–10.7), low serum levels of HDL cholesterol (OR/95% CI: 5.1/3.6–7.4). Haplogroup frequencies in obese and nonobese subjects are reported in Table 2. All nine common European haplogroups (H, I, J, K, T, U, V, W, and X) [21] were identified in most of our subjects (94.4%), whereas the R, L, N, B, and F haplogroups (collectively indicated as “others” in Table 2) were rare (5.6%). European haplogroup frequencies were very similar in our obese and nonobese subjects, and the H haplogroup was the most frequent (about 40% of subjects). The T (*P* = 0.004) and J (*P* = 0.02) haplogroup frequencies were higher and lower, respectively, in obese subjects than in controls (haplogroup T: 13.2% versus 6%; haplogroup J: 5% versus 9.7%). At the *χ* ^2^ test, subjects with the T haplogroup had an increased risk of about two-fold for obesity (OR/95% CI = 1.94/1.16–3.22; *P* = 0.004), whereas subjects with the J haplogroup were less exposed to obesity (OR/95% CI = 0.63/0.45–0.89; *P* = 0.02) than subjects with the other haplogroups. These findings were confirmed by the results of bootstrap analysis based on 100 bootstrap samples.

When we divided our populations into males and females, the prevalence of the T and J haplogroups remained significantly higher and lower, respectively, in the obese females (12.4% versus 4.9%, OR/95% CI = 2.18/1.01–4.39; *P* = 0.012 for haplogroup T; 4.7% versus 11.9%, OR/95% CI = 0.55/0.38–0.78; *P* = 0.009 for haplogroup J). In addition, the T haplogroup was correlated to the degree of obesity. In fact, this haplogroup was significantly more frequent in obese subjects with a BMI higher than in those with a BMI lower than the median BMI (45 kg/m^2^) (16% versus 10%, resp.; *P* = 0.04). Binomial logistic regression analysis (dependent variable: median BMI and independent variables: clinical and biochemical investigated parameters) showed that haplogroup T and systolic blood pressure are risk factors for a high degree of morbid obesity, namely, BMI > 45 kg/m^2^ (OR/95% CI: 2.3/1.3–4.3 and OR/95% CI: 1.1/1.02–1.4, resp.); in fact together they account for 8% of the BMI. We did not find an association between mtDNA haplogroups and the clinical/biochemical tested variables, or in haplogroup frequencies according to MS presence/absence.

## 4. Discussion 

Mitochondrial DNA haplogroups have been associated with metabolic disorders in various populations [3, 22, 23], although not yet in morbid obese Caucasians from southern Italy. The prevalence of MS in our obese patients (41%) was similar to that previously reported in a multicenter Italian study (43.6%) [24]. We found that haplogroup H was the most common haplogroup in both obese and control subjects (46.6% and 40.7%, resp.) from southern Italy, which is in agreement with the H frequency reported in other European populations (haplogroup H frequency: 13.3%–41.7%) [21]. We also show that haplogroups T and J conferred an increased and decreased risk for obesity, respectively. The J haplogroup was reported to protect against the onset of diseases related to oxidative stress and/or low inflammation grade, such as ischemic cardiomyopathy [25]. In fact, subjects bearing this haplogroup showed lower oxygen consumption (because the lower efficiency of the electron respiratory chain resulted in reduced production of radical oxygen species) than those with the H haplogroup [25]. 

On the other hand, subjects with haplogroup T could be intrinsically prone to develop defects in the mitochondrial oxidative phosphorylation system, which, in turn, negatively affects the performance of mitochondrial ATP production [11]. In fact, haplogroup T was reported to be more frequent in white men with fertility problems (a condition strongly dependent on ATP production) [13] and among Spanish patients affected by left ventricular hypertrophy [26]. Indeed, haplogroup T was less frequent among elite endurance athletes (possibly related to a negative effect on cardiac adaptation to endurance training) [11]. Amo et al. [27] reported that mitochondrial bioenergetic capacities and coupling efficiencies in cultured carcinoma cells did not differ between transmitochondrial cybrids harbouring mitochondria with haplogroup H and those harbouring mitochondria with haplogroup T [27]. However, the authors suggest that this result could be partially inconclusive because the effect of mitochondrial haplogroups might be too small to be detected by the procedures used [27], or they might need particular nuclear genes in order to be expressed, or they might need additional signals (nutrients and/or oxidative stress molecules) to influence oxidative phosphorylation functions, as previously described for the Uk haplogroup [28]. The foregoing studies suggest that, in our obese patients, the T haplogroup could contribute to affect the mechanisms of energy balance regulation so leading to increased fat depots.

In conclusion, our finding that haplogroup T increases the risk of obesity by about two-fold suggests that, besides nuclear genome variations and environmental factors, the T haplogroup contributes to morbid obesity in our study population from southern Italy.

## Figures and Tables

**Table 1:** General and biochemical characteristics of the morbid obese patients studied (*n* = 500).

	Median	Percentiles	
		2.5th	97.5th
Age (years)	32.59	17.50	57.00
Height (m)	1.66	1.51	1.85
Weight (kg)	125.54	98.00	178.40
BMI (kg/m^2^)	45.10	38.20	65.40
Waist circumference (cm)	131.00	106.00	170.00
Hips circumference (cm)	134.00	114.90	158.10
W/H ratio	0.99	0.82	1.19
RQ	0.87	0.74	1.01
BMR (kcal)	2237.50	1581.38	3278.63
FFM (%)	51.40	40.28	64.11
FM (%)	48.55	35.89	59.73
SBP (mmHg)	124.45	100.00	160.00
DBP (mmHg)	80.00	60.00	100.00
Heart rate (beats/min)	80.00	60.00	100.00
Glucose (mmol/L)	5.05	3.89	7.53
Total cholesterol (mmol/L)	4.75	3.11	7.12
HDL cholesterol (mmol/L)	1.21	0.73	1.82
Triglycerides (mmol/L)	1.42	0.56	3.16
AST (U/L)	23.00	13.55	64.45
ALT (U/L)	31.00	12.00	84.45
GGT (U/L)	26.00	10.00	87.50
CHE (U/mL)	10066.37	5828.90	15084.55
Total bilirubin (*µ*mol/L)	9.41	4.28	20.94
Uric acid (mmol/L)	0.35	0.21	0.57
Albumin (g/dL)	4.30	3.60	4.90
Total Protein (g/dL)	7.56	6.76	8.30
Creatinine (*µ*mol/L)	70.72	44.20	106.08
Urea (mmol/L)	5.33	3.33	8.16
ALP (U/L)	76.00	42.75	247.75
C-peptide (ng/mL)	3.90	1.80	7.35
Insulin (mU/L)	19.00	6.90	53.03
HOMA	4.26	1.43	14.66

ALP: alkaline phosphatase; ALT: alanine transaminase; AST: aspartate transaminase; BMI: Body Mass Index; BMR: basal metabolic rate; CHE: cholinesterase; DBP: diastolic blood pressure; FFM: fat-free mass; FM: fat mass; GGT: *γ*-glutamyl transferase; HOMA: homeostatic model assessment; RQ: respiratory quotient; SBP: systolic blood pressure; W/H: waist/hip.

**Table 2:** Haplogroup frequencies in morbid obese patients (*n* = 500) and controls (*n* = 216).

Haplogroup	Obese patients *n* (%)	Control subjects *n* (%)	*P*	OR/95% CI
H	233 (46.6)	88 (40.7)		
I	7 (1.4)	6 (2.8)		
J	25 (5.0)	21 (9.7)	0.02	0.63/0.45–0.89
K	29 (5.8)	18 (8.3)		
T	66 (13.2)	13 (6.0)	0.004	1.94/1.16–3.22
JT	0 (0)	1 (0.5)		
U	85 (17.0)	39 (18.1)		
V	13 (2.6)	4 (1.9)		
W	8 (1.6)	2 (0.9)		
X	10 (2.0)	8 (3.7)		
Others	24 (4.8)	16 (7.4)		
